# Pediatric and Adolescent T-type Distal Humerus Fractures

**DOI:** 10.5435/JAAOSGlobal-D-17-00040

**Published:** 2017-11-01

**Authors:** Charles A. Popkin, Katherine A. Rosenwasser, Henry B. Ellis

**Affiliations:** From the Department of Orthopedic Surgery, New York Presbyterian, Columbia University Medical Center, New York, NY (Dr. Popkin, Dr. Rosenwasser), and the Department of Orthopaedic Surgery, University of Texas Southwestern, Children's Medical Center, Texas Scottish Rite Hospital for Children, Dallas, TX (Dr. Ellis).

## Abstract

Although fractures of the elbow are extremely common in pediatric patients, the T-type distal humerus fracture is rare and offers unique challenges. The mechanism of injury may be similar to the adult counterpart and is usually caused by a fall onto a flexed elbow or from a direct blow. Diagnosing these injuries may be difficult. They often resemble extension-type supracondylar fractures, yet the treatment algorithm is quite different. In younger patients, percutaneous pinning remains a viable option, but for older adolescents, open reduction and internal fixation provides stable fixation at the elbow and the most reliable restoration of the articular surface. Appropriate imaging, careful radiographic diagnosis, and choice of surgical technique are of paramount importance when treating young patients with this injury. Most pediatric and adolescent patients with T-type distal humerus fractures have results better than those of adults but often worse than other elbow fractures in this age group.

Although injuries to the upper extremity in children are very common, T-type distal humerus fractures are more rare, representing <2% of observed pediatric elbow fractures.^1,2,3^ The typical age range of other common elbow fractures in the pediatric population tends to occur at younger ages (ie, 5–8 years old). T-type distal humerus fractures are more common in the adolescent population (ie, >10 years old) and are not typically seen in the younger age group.^4–5^ Boys tend to be affected more than girls. These injuries often result from a fall onto the posterior aspect of a flexed elbow, with the nondominant arm affected at a ratio of 2.5:1.^6^ A high degree of vigilance is required to diagnose intra-articular extension in these fractures because most require open surgical management.^3^

## Anatomy

**Figure 1:**
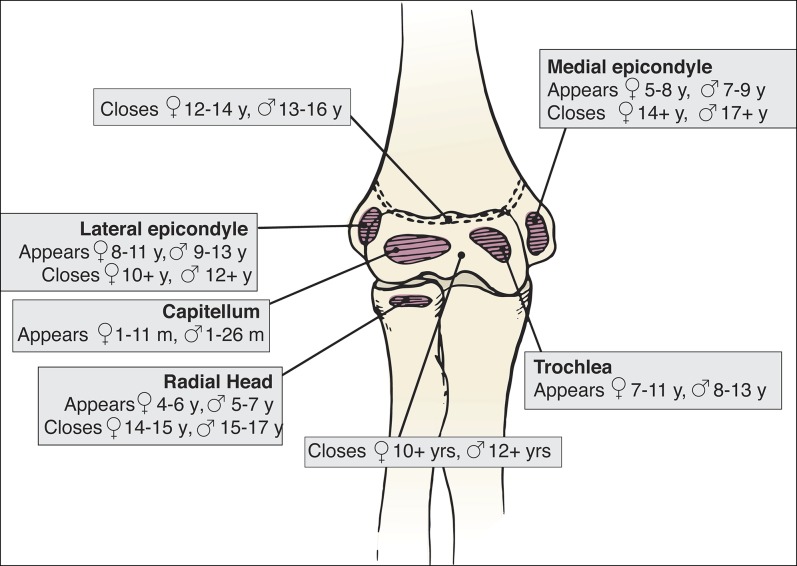
Image of the ossification centers of the elbow. The age in which these ossification centers appear and fuse are noted for both girls and boys.

The complexity of the chondro-osseous development of the pediatric elbow must be considered when treating any skeletally immature elbow injury. T-condylar and other elbow fractures, including transphyseal, lateral condyle, medial epicondyle, and supracondylar fractures, require knowledge of six secondary ossification centers around the elbow and their variable timing of ossification and closures^7,8^ (Figure 1). Although the initiation of ossification typically occurs at younger ages, the time to fusion occurs during early and late adolescence and has clinical application in the T-condylar distal humerus fracture pattern. Comminuted fractures typically involve these secondary ossification centers.

The newborn has a completely cartilaginous distal humeral epiphysis, but there is ossification of the metaphysis at birth.^9^ Between the age of 6 months when the capitellum begins to ossify and 14 to 18 years when the medial epicondyle completes fusion, the pediatric elbow is susceptible to a wide variety of fracture patterns when subjected to trauma.^10^ While children are nearing completion of growth, the capitellum, lateral epicondyle, and trochlea fuse to form one epiphyseal center. This fused epiphysis then fuses with the distal humeral metaphysis during age 12 to 16 years.^9^

Even before complete fusion, the distal humerus chondral surface is made up of two articulated surfaces that must be appreciated during fixation: the trochlea for the ulnohumeral articulation and the capitellum for the radiocapitellar articulation. The humeral epiphysis, when skeletally mature, has both rotational and angular relationships to the humeral shaft (Table 1). These values are an important consideration in both open and closed reductions of any elbow injury. Failure to restore these relationships may cause cosmetic or functional malunions. Knowledge of the proximity of neurovascular structures is important with this injury (Table 1); however, these references should be used with caution in the pediatric elbow. Their relationship in the immature elbow may be closer than the listed references.

**Table 1:**
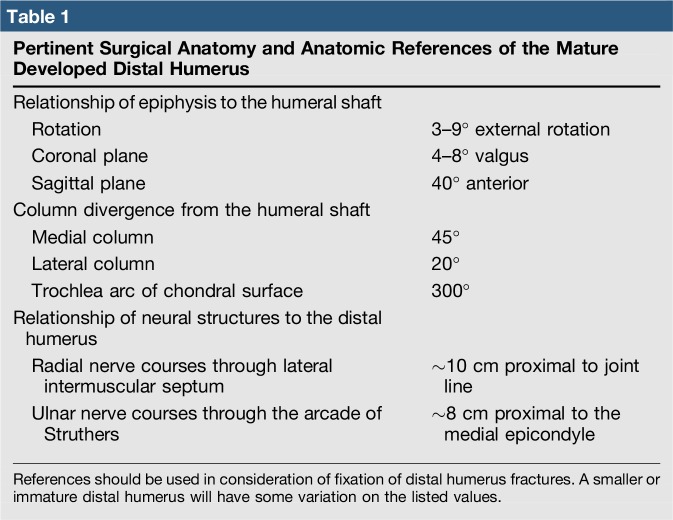
Pertinent Surgical Anatomy and Anatomic References of the Mature Developed Distal Humerus

## Mechanism of Injury

Similar to mechanisms that produce the T-type fracture pattern in adults, this injury is mostly caused by an axial load onto an outstretched arm or a direct blow to the flexed elbow.^11^ In a series of 17 pediatric and adolescent T-condylar humerus fractures, Re et al^6^ reported that nine fractures (53%) were from a pedestrian fall; seven (42%) were from high-energy mechanisms, including from a bike (3), skiing (2), motor vehicle accident (1), and skateboard (1).

These variable modes of injury may correlate with different contractions of the elbow flexors and extensors that accentuate the coronal plane separation of the fracture fragments. In injuries obtained in elbow flexion, the apex of the trochlea may serve as a wedge and cause the condylar fragments to lie anterior to the shaft. In the extension variant, however, the coronoid portion of the semilunar notch wedges the condyles, forcing them posteriorly.^12^ During the fall, the semilunar notch or coronoid process of the olecranon may act as a wedge, splitting the trochlea into the characteristic T-shaped pattern between the medial and lateral condyles.^12^

## Classification

**Figure 2:**
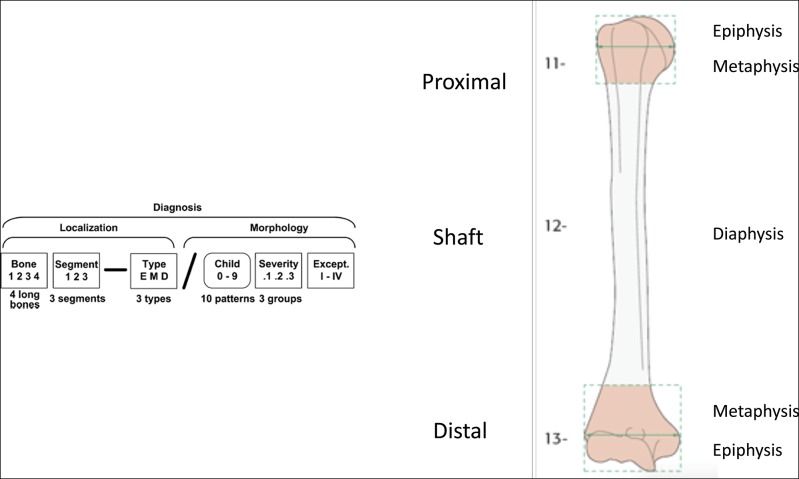
Pediatric OA/OTA classification of distal humerus fractures. E = epiphysis, M = metaphysis, D = diaphysis

The most commonly used classification system for adolescent T-type distal humerus fractures is the adult OA/OTA classification (Figure 2). Some have called into the question the utility of this OA/OTA adult type classification system because of the complexities of the chondro-osseous development in the growing child. In 2006, a pediatric OA/OTA classification was developed and validated for skeletally immature long bone pediatric fractures.^13^ This pediatric classification distinguishes between the epiphysis (E), metaphysis (M), and diaphysis (D) and accounts for common fracture patterns specifically seen in the skeletally immature patient (Figure 2). The pediatric OA/OTA classification for the T-condyle distal humerus fracture abides by the same principles as the adult classification. The pediatric OA/OTA classification for this fracture is 13-E/4.2, without a subgroup for the severely comminuted distal humerus fractures, as these are extremely rare in the skeletally immature patient. Other than as a descriptor, there does not appear to be therapeutic or prognostic value to this classification.

Less commonly used is the Toniolo and Wilkins classification that groups these fractures into three types: type I, minimally displaced fractures; type II, displaced but not comminuted; and type III, comminuted fractures. Adolescent patients are generally classified the same as adults, with the OA/OTA classification system being most widely used.^14^

## Clinical Features

Patients most often present with pain and swelling around the elbow, preferring to hold the upper extremity in a semiextended position. The elbow may appear ecchymotic or deformed, similar to the appearance of a supracondylar humerus fracture. Refusal to move the arm is common, and many children will be unwilling to have the extremity ranged or examined.

While permanent nerve damage during the injury itself is not exceedingly common, a careful neurovascular examination is critical because these injuries tend to occur with higher energy mechanisms, as transient traction neurapraxia may be more common.^15^ Between 29% and 38% of pediatric and adolescent fractures present with paresthesias in the ulnar nerve distribution; about a fourth of these will also have intrinsic weakness in the hand.^6,16^ The presence of an ulnar nerve palsy may be more likely to appear when the medial epicondyle is fractured. Radial nerve injuries and brachial artery injuries have been reported but are extremely rare.^6^ Furthermore, a recent study highlighted a correlation between obesity and more complex pediatric elbow fracture patterns, such as the T-type, demonstrating that these patients also had higher pre- and post-operative nerve palsies.^17^

## Imaging

**Figure 3:**
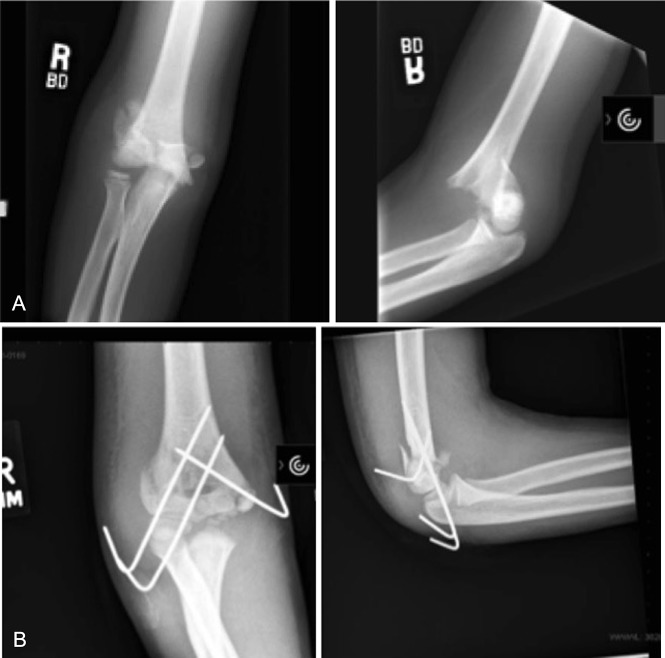
**A,** AP and lateral radiographs of a 9-year-old boys with a T-type distal humerus fracture. On initial glance, it resembles a regular extension-type supracondylar humerus fracture. **B,** AP and lateral radiographs after percutaneous fixation of the pediatric T-type distal humerus fracture.

Standard radiographs of the elbow, including AP and lateral views, should be obtained. Oblique images may be beneficial to view minimally displaced fractures. Displacement of condylar fractures that include the lateral condyle and medial epicondyle fractures are best visualized on an internal oblique radiograph.^18,19^ Radiographically, these fractures may closely resemble extension-type or low supracondylar fractures (Figure 3). Careful attention must be paid when reading these radiographs, as the vertical fracture line can be easily missed, and the distal humerus may still be largely cartilaginous, especially in younger children.^20^ Historically, traction radiographs have been used to provide more information regarding the vertical split fracture line, but this has fallen mostly out of favor because of the difficulty of pediatric patients tolerating the examination.

**Figure 4:**
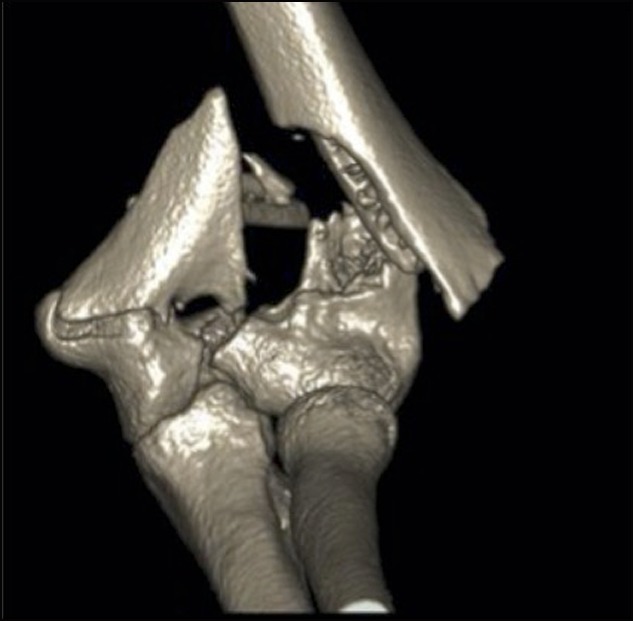
Three-dimensional CT scan in a 15-year-old boy with a T-type distal humerus fracture, to aid with preoperative planning.

Advanced imaging is rarely warranted in the diagnosis of this injury, but there may be a role for a CT scan, especially with 3D images for preoperative planning purposes or if it is thought that the fracture is not clearly elucidated on radiographs alone (Figure 4). If an intra-articular segment of a supracondylar humerus fracture in a pediatric elbow injury is suspected, a CT scan may allow for proper planning for treatment and also in pin placement. In patients who are younger than 10 years, an MRI or a CT arthrogram may be advantageous to visualize the unossified epiphysis and its fracture displacement. However, an MRI or CT arthrogram may be difficult to obtain in this age group without the use of sedation.

Some authors also advocate for arthrograms, especially in young children, if the diagnosis remains questionable.^12^ In pediatric fracture patterns, an arthrogram has utility after percutaneous fixation of a nondisplaced intra-articular splint while in the operating room using fluoroscopy. Anatomic reduction of the chondral surface is difficult to visualize in an immature elbow.

## Management Options

### Nonsurgical

Although nonsurgical management is often a mainstay in the treatment of pediatric trauma, there is a minimal role for closed reduction and casting in this subclass of fractures.^21^ Good results have been reported for truly nondisplaced fractures with cast immobilization, achieving full elbow range of motion and fracture union. However, these tend to occur in the younger population, likely secondary to a thicker periosteum and elasticity of the articular cartilage, resulting in less displacement of fracture fragments. Other arguments for nonsurgical management include fractures with less soft-tissue stripping and vascular disruption, resulting in a lower likelihood of osteonecrosis and growth disturbance.^20^

### Surgical

The decision of when and how to operate on T-type distal humerus fractures is largely related to the degree of articular displacement. Indications for urgent surgical treatment include open fractures, skin tenting, or neurovascular compromise. With the distal fragment in extension, the proximal spike of the humerus may tether neurovascular structures across the elbow. Thus, prompt awareness of fracture pattern and physical examination may lead the surgeon to a more urgent surgical treatment to preserve the neurovascular bundle.

## Percutaneous Pinning

Many surgeons recommend treatment of T-type distal humerus fractures with percutaneous Kirschner wire (K-wire) fixation in cases of younger patients with minimal displacement.^22^ The decision to use this technique relies on the surgeon's confidence in maintaining the integrity of the medial and lateral columns. Thickened periosteum in the skeletally immature patient can further assist with fracture stability and, thus, allows for percutaneous fixation.

Some series describe the use of this technique, even in displaced fractures, citing improved complication rates with regard to iatrogenic nerve palsy, heterotopic ossification, and infection with comparable functional results.^23^ The reoperation rate is low with this technique, as pins are able to be removed in the outpatient setting. Kanellopoulous and Yiannakopoulos^11^ describe a technique in which partially threaded pins are used for interfragmentary compression, and then flexible intrameduallary nails are inserted to stabilize the supracondylar portion of the fracture. In this report of two patients, both patients went on to union and were able to return to high-level athletics. This minimally invasive approach may be appropriate in preadolescent patients with thick periosteum and minimal fracture displacement. This approach avoids the morbidity of an open approach by eliminating posterior elbow dissection, potentially minimizing elbow stiffness and the risk of osteonecrosis of the distal humerus.^14,24^ In minimally displaced T-type fractures, there may be a role for the percutaneous approach, as evidenced by the results of several small studies.^22,23^

### Technical Considerations for Percutaneous Fixation

Consideration of percutaneous pinning does not negate preparation for open reduction and internal fixation. The surgical consent and discussion with the family should include the possibility of an open procedure if necessary and the use of contrast for an intra-articular arthrogram to confirm reduction. Documentation of preoperative neurovascular examination is also recommended because of the potential for iatrogenic injury to the ulnar nerve.

**Figure 5:**
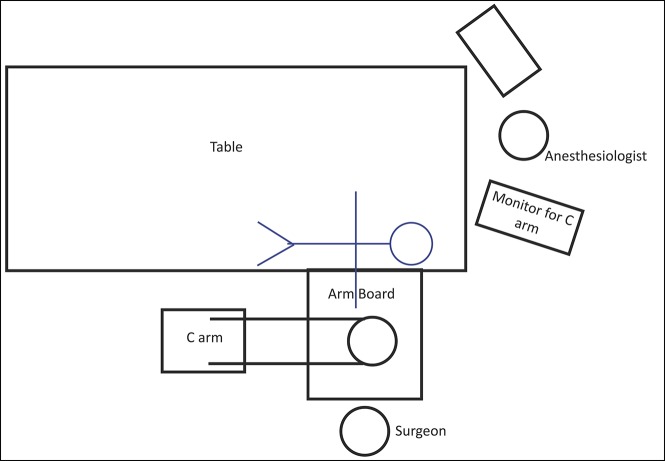
Image depicting preferred room setup for the C-arm and monitor for the cases.

Room position and equipment should also be prepared or be available for an open conversion. If a closed reduction is attempted, K-wires (2 mm), both threaded and smooth-tip, can be used. Setup of the room should allow for the C-arm to be positioned parallel to the bed, coming in from the foot of the bed. This will allow for the device to arc and provide lateral images without rotating the arm during the reduction and/or fixation (Figure 5). The position should allow for the complete extremity to be on the radiolucent arm board for adequate imaging. The display screen for the fluoroscopic images can be placed at the head of the patient (Figure 5).

Reduction maneuvers depend on fracture type and location of the intra-articular split. Provisional fixation of the condyles in a minimally displaced intra-articular fracture will allow for a reduction maneuver of the fragment to the humeral shaft. Extended fragments are commonly reduced with manual anterior force on the olecranon while simultaneously providing axial tension and flexion at the elbow. Flexed fragments are reduced with the arm at 90° of flexion and a posterior force applied to the forearm.

Optimal pin placement will help avoid loss of reduction or the need for revision procedures.^25^ If indicated, a single cross pin parallel to the joint surface and perpendicular to the fracture will give provisional stability. Lateral-to-medial pins are typically preferred to avoid damage to the ulnar nerve. Confirmation of penetration through the medial humeral cortex is required for stable fixation. Divergence of the pins at the fracture site also improves stability.^26^

Medial-to-lateral pins are often necessary but may put the ulnar nerve at risk if the surgeon is not diligent in protecting the ulnar nerve. Medial pins are ideally placed with the elbow at <90° of flexion to avoid anterior displacement of the ulnar nerve during higher flexion angles. As opposed to a true percutaneous pin placement, we recommend making a small incision anterior to the palpable medial epicondyle. This is followed by blunt dissection to the medial epicondyle. A soft-tissue protector is then used to avoid ulnar nerve damage during advancement of the pin. Iatrogenic injury to the ulnar nerve from medial pin placement has been reported to range from 1.4% to 15.6%.^27^

### Open Reduction and Internal Fixation

Various options exist for both approach and fixation when managing this fracture, without clear evidence available for the safest or most effective. Early studies described a lateral approach to this injury but showed unsatisfactory results with flexion contractures, growth disturbance, and lack of visualization of the ulnar nerve, leading most surgeons to use a posterior approach, as reflected in the current literature.^14^

#### Approach

The decision on which approach to use for the treatment of these fractures is multifactorial and based on a combination of surgeon preference, surgeon comfort, and comminution of the fracture. For simpler fracture patterns with less comminution and/or displacement, a triceps-sparing or -splitting approach may be generally favored. More complex patterns may necessitate a more extensive exposure that requires a triceps slide (Bryan-Morrey) or an olecranon osteotomy.^3,14,28,29^

Advantages and disadvantages of each approach are, in part, based on the comfort level and experience of the surgeon. A Cheung and Steinmann^30^ is also an excellent resource for the different surgical approaches related to the distal humerus and elbow.

#### Triceps Splitting

A commonly used surgical approach involves a longitudinal splitting of the triceps.^28,31^ This approach consists of a posterior curvilinear incision over the elbow, with division of the triceps tendon while maintaining its attachment to the olecranon. The incision through the muscle may then be extended proximally, with retraction of the triceps both medially and laterally to provide additional exposure. Most surgeons choose to isolate and protect the ulnar nerve during this approach to minimize iatrogenic injury.^15^ Proponents of this approach argue that it reduces the degree of soft-tissue stripping that is required for fracture visualization, affords adequate exposure of the articular surface, and does not have a great deal of morbidity or loss of extensor strength.^32^

#### Bryan-Morrey (Posteromedial Approach)

An alternative approach that has been described similarly avoids osteotomizing the proximal ulna but spares the triceps by reflecting it laterally during exposure of the distal humerus. This approach uses a 10- to 15-cm extensile curvilinear incision over the olecranon with identification of the ulnar nerve. Its hallmark, then, is to elevate the medial aspect of the triceps along the intramuscular septum to the posterior capsule, all as a single sleeve of muscle, fascia, and periosteum that may be repaired after fracture fixation.^33^ Proponents of this approach suggest that it offers comparable joint exposure and protection of the ulnar nerve while minimizing disruption to the triceps muscle and, thus, the extensor mechanism. Case series have shown no significant difference in triceps strength or range of motion postoperatively between this approach and the conventional triceps split.^34^

#### Olecranon Osteotomy

**Figure 6:**
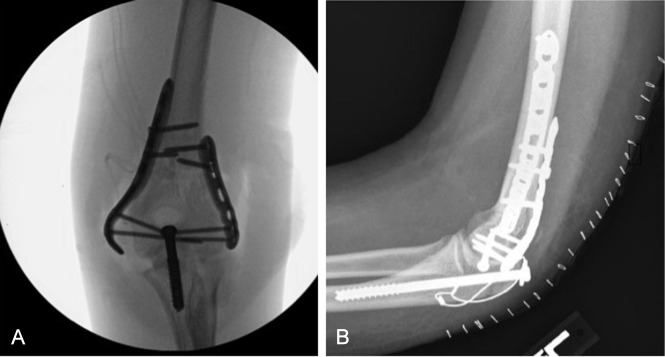
**A,** AP radiograph of a 13-year-old boy who fell from a ladder and sustained a T-type fracture. A large fragment screw alone was used for fixation of the olecranon osteotomy. **B,** Lateral radiograph in a 14-year-old girl injured in all-terrain vehicle rollover. A large fragment screw and tension band were used for fixation.

Although rare in the adolescent population, more complex cases with increasing degree of articular comminution may indicate an olecranon osteotomy approach to improve access to the joint surface.^3^ This approach uses a paratricipital approach to the posterior humerus and then a chevron-shaped osteotomy in a technique similar to that used in adult fractures.^28^ Other surgeons prefer a transverse or oblique olecranon osteotomy. Drilling and tapping the olecranon before the osteotomy in preparation for screw fixation after fracture fixation is advantageous. The ulnar nerve may either be dissected and retracted or transposed, according to the surgeon's preference. After fracture fixation, the osteotomy site is repaired with double K-wires and tension banding.^3^ However, the traditional tension band technique has been associated with a high complication rate.^35,36,37^ Other olecranon fixation techniques include a plate with screws, a single large partially threaded screw, or a screw with a tension band. A plate-and-screw construct for fixation of the olecranon osteotomy is more expensive and has shown a small incidence of skin necrosis and a high incidence of symptomatic instrumentation. Many pediatric orthopaedic surgeons prefer a single partially threaded screw to fix the osteotomy (Figures 6). Adding a tension band to the intramedullary partially threaded screw allows for higher forces to be applied to the triceps and, perhaps, early motion.^38^ If symptomatic, the olecranon instrumentation can be removed 4 to 6 months after surgery following evidence of radiographic union.^3^ Complications may result from this technique, as well, including nonunion, symptomatic instrumentation, and capsular adhesions.

#### Fixation

A myriad of fixation strategies need to be considered before starting the case. Provisional fixation of comminuted fragments may occur with a 2-mm K-wire or a 2.7-mm cortical screw before definitive fixation. Headless compression screws or pins should be available if osteochondral fixation is needed. When severe bone loss is present in the trochlea, autologous iliac crest may be considered. The first priority in these cases is to reduce anatomically the joint surface, usually with a single transverse intercondylar screw.

Secondarily, the integrity of the medial and lateral columns must be restored, using a neutralization plating technique. Internal fixation strategies tend to be more widely agreed upon, with most surgeons preferring interfragmentary screws and dual-column plating, most often medially and laterally, either in parallel or in a perpendicular orientation.^6^ Dual-plate fixation is recommended for adolescent or skeletally mature patients but may not be needed for the younger elbow because the thickened periosteum of the distal humerus may allow for pin-and-screw fixation after direct reduction. This may help avoid prominent instrumentation in the immature elbow injury that may require device removal at the risk of suboptimal fixation.

In most pediatric or adolescent cases, both 3.5-mm and 2.7-mm reconstruction plates should be available for fixation. Reconstruction (ie, recon) plates are ideal because they can be contoured to match the columns in pediatric patients of variable sizes. Precontoured plates are also available for skeletally mature patients. Precontoured plates for T-condylar fractures are not currently designed for the pediatric elbow.

## Rehabilitation

Many surgeons advocate for early range of motion using a continuous passive motion (CPM) machine within the first few days postoperatively, whereas others cast patients for several weeks for soft-tissue rest. Studies have shown that CPM therapy may improve terminal flexion, but it does not seem to affect extension.^6^ However, early range of motion does seem to provide patients with more functional elbow range of motion sooner than those who are immobilized.^5^ Patients who are fixed using either a triceps-splitting or triceps-sparing approach show improved strength and range of motion about the elbow with early motion.^32,34^ This early rehabilitation may improve motion in only the short term, as a Beck et al^5^ study found similar outcomes at 1-year follow-up in patients who started therapy late.

## Outcomes

Compared with other pediatric elbow fractures, results with T-type fractures are poorer, with an increased rate of complications and reduced range of motion, but are better than those of the adult population with a T-type fracture.^14^ Other pediatric elbow fractures are rarely treated with an open procedure and arthrotomy and thus do not have the difficulties with stiffness and wound complications, as do some of the T-condylar humerus fractures. Also, when internal fixation is required, it often requires removal of symptomatic devices.^16^ A possible cause of reduced outcomes may be the higher energy of injury required to sustain a T-condylar fracture compared with a typical pediatric elbow fracture.

However, improved outcomes, compared with those of adults, may be a result of residual remodeling, even in older patients, as the trochlea continues to fuse until the mid to late teen years.^3^ Elbow stiffness, nerve injury, and heterotopic ossification are worse after open reduction and internal fixation than with closed reduction and percutaneous pinning. In a systematic review by Anari et al,^14^ patients lose approximately 11° of extension but maintain normal flexion, with younger patients (younger than age 10 years) regaining more function than do adolescent patients. In addition, the authors found no independent risk factors for stiffness or elbow function based on surgical approach, although outcome scores were highest in patients with a triceps-splitting approach.^14^ Cook et al^16^ found no difference in outcome based on the surgical approach. Another study showed that regardless of the approach, the biggest indicator for loss of range of motion postoperatively was the degree of preoperative articular damage.^6^

## Complications

Similar to their adult counterparts, the most common complication for young patients after T-type distal humerus fractures is elbow stiffness.^23^ This is partially due to significant soft-tissue trauma at the time of injury and iatrogenic manipulation. A review of T-type fractures in adolescents reported that 16.5% of patients reported notable elbow stiffness at the final follow-up.^14^ Factors that contribute to this loss include the degree of intra-articular displacement and comminution at the time of the injury as well as the triceps-splitting approach, causing the most postoperative stiffness.^6^ This is thought to be secondary to scar formation in the olecranon fossa that may limit extension as well as triceps muscle adhesions that may limit flexion.

Another potentially morbid complication after this injury is transient neuropathy, most commonly manifested as ulnar paresthesias. A combination of the adult and pediatric literature would suggest a range from 10% to 44% incidence of immediate or delayed transient ulnar neuropathy after open reduction and fixation, with variation seen in different approaches to expose the intra-articular reduction.^15,39,40^ These data suggest that careful handling of the ulnar nerve is critical during fixation.

Historically, when an open approach is used for a T-condylar humerus fracture, an ulnar nerve transposition was generally recommended.^41,42^ However, recent studies in the adult population have suggested that an ulnar nerve transposition is not protective against neuropathy or neuritis and that an ulnar nerve decompression is equally effective.^40,42^ Chen et al^42^ compared distal humerus fractures that had an ulnar nerve transposition with those that did not. At an average follow-up of 9.6 months, the incidence of ulnar neuritis was four times higher in those who underwent an ulnar nerve transposition. An ulnar nerve decompression without a transposition has been validated to improve ulnar nerve symptoms.^43^ These studies were conducted in an adult population, so some caution should be taken in extrapolating to the pediatric population. However, although some fracture patterns may dictate the need for an ulnar nerve transposition, most of these injuries may require only a careful ulnar nerve dissection and decompression.

Reoperation after open reduction internal fixation is most often attributed to painful hardware, formation of heterotopic bone, deep infection, and failure of fixation, typically associated with the use of the olecranon osteotomy approach.^44,45^ Growth disturbance is not common, as these fractures tend to occur in older children or adolescents, and the distal humeral physis contributes only 20% of the growth of the arm.^46^

## Summary

T-type distal humerus fractures represent an unusual fracture pattern in the pediatric patient. These fractures are often difficult to diagnose, given the large cartilaginous component remaining in the young elbow, and may be confused with extension-type supracondylar fractures. Identification of this injury is critical because nonsurgical management generally does not lead to satisfactory outcomes except in nondisplaced fractures. Open reduction with internal fixation is the mainstay of management in patients of nearly all age groups, with the main goals being anatomic congruity of the joint and restored integrity of the medial and lateral columns. For less articular displacement, the triceps-splitting or triceps-sparing approaches are preferred, with the latter showing slightly better postoperative range of motion but similar extensor strength. For complex or comminuted articular reductions, the olecranon osteotomy approach may be necessary. Most common complications include elbow stiffness and transient neurapraxia. Because of the rarity of this injury, no consensus exists on ideal treatment, as yet. Further study is needed to optimize management and rehabilitation recommendations and to minimize complications.
